# Maternal Embryonic Leucine Zipper Kinase Promotes Tumor Growth and Metastasis via Stimulating FOXM1 Signaling in Esophageal Squamous Cell Carcinoma

**DOI:** 10.3389/fonc.2020.00010

**Published:** 2020-01-28

**Authors:** Liang Chen, Qiuren Wei, Shuning Bi, Songqiang Xie

**Affiliations:** School of Pharmacy, Henan University, Kaifeng, China

**Keywords:** ESCC, MELK, tumorigenesis, metastasis, FOXM1

## Abstract

Esophageal squamous cell carcinoma (ESCC) is a common gastrointestinal malignancy and is one of the most important cause of cancer related mortalities in the world. However, there is no clinically effective targeted therapeutic drugs for ESCC due to lack of valuable molecular therapeutic targets. In the present study, we investigated the biological function and molecular mechanisms of maternal embryonic leucine zipper kinase (MELK) in ESCC. The expression of MELK mRNA and protein was determined in cell lines and clinical samples of ESCC. MTT, focus formation and soft agar assays were carried out to measure cell proliferation and colony formation. Wound healing and transwell assays were used to assess the capacity of tumor cell migration and invasion. Nude mice models of subcutaneous tumor growth and lung metastasis were performed to examine the function of MELK in tumorigenecity and metastasis of ESCC cells. High expression of MELK was observed in ESCC cell line and human samples, especially in the metastatic tumor tissues. Moreover, overexpression of MELK promoted cell proliferation, colony formation, migration and invasion, and increased the expression and enzyme activity of MMP-2 and MMP-9 in ESCC cells. More importantly, enhanced expression of MELK greatly accelerated tumor growth and lung metastasis of ESCC cells *in vivo*. In contrast, knockdown of MELK by lentiviral shRNA resulted in an opposite effect both *in vitro* and in animal models. Mechanistically, MELK facilitated the phosphorylation of FOXM1, leading to activation of its downstream targets (PLK1, Cyclin B1, and Aurora B), and thereby promoted tumorigenesis and metastasis of ESCC cells. In conclusion, MELK enhances tumorigenesis, migration, invasion and metastasis of ESCC cells via activation of FOXM1 signaling pathway, suggesting MELK is a potential therapeutic target for ESCC patients, even those in an advanced stage.

## Introduction

Esophageal cancer (EC) is one of the most common gastrointestinal malignancies and ranks as the sixth most important cause of cancer mortalities globally, with an estimated 509,000 new deaths every year (1, 2). As one of the most common histologic subtypes of EC, ESCC usually composes over 90% of all EC cases in parts of Asia and Sub-Saharan Africa (1, 3). Although remarkable advances in surgical treatments, chemotherapy, and radiotherapy for ESCC, the 5-year overall survival rate is still <20%, due to the high incidences of tumor metastasis (4, 5). Recent advances demonstrated that understanding the molecular mechanisms of malignant development and progression is very important for the development of novel targeted therapeutic agents for the treatment of human cancer (6). However, up to now, ESCC is still one of the least studied malignancies in the world, and the critical signaling pathways and molecular mechanisms involved in the initiation, development and progression of ESCC are not fully elucidated (7). Therefore, there is an urgent need to identify the potential molecules involved in ESCC tumorigenesis and metastasis.

MELK is a serine/threonine kinase that belongs to the member of the AMP-activated protein kinase (AMPK)/sucrose non-fermenting kinase 1 (SNF1) family (8). Unlike most members of this family, MELK is not involved in cellular energy metabolism balance (9). MELK, also known as murine MPK38, was originally discovered by Heyer et al. as a signal transduction factor and was predominantly expressed in the mouse egg and preimplantation embryo (10). Simultaneously, Gil et al. found that MELK was mainly expressed in T lineage and macrophage/monocyte cells of thymus and spleen tissues, suggesting an important role of this gene product in hematopoietic cell function and development (11). Subsequently, mounting evidence demonstrated that MELK plays a crucial role in diverse cellular processes including cell cycle progression, mitosis, proliferation, RNA processing, and spliceosome assembly (12). High expression of MELK was observed in less differentiated human acute myeloid leukemia (AML) cell lines and AML blasts with complex karyotypes and was associated with worse clinical outcome (13). Moreover, MELK overexpression was also found in chronic lymphocytic leukemia (CLL) cells, and positively correlated with advanced stage, higher WBC count, increased β2-MG level, elevated LDH, unmutated IGHV, deletion of 17p13, positive ZAP-70 and inferior prognosis of CLL patients (14). Besides hematologic malignancies, mounting evidence showed that MELK is frequently up-regulated in a wide range of malignant solid tumors. By using bioinformatic and oligonucleotide microarray analyses to compare gene expression between human normal and tumor tissues, Gray et al. provided evidence that MELK expression was elevated in 13, 23, and 96% of ovarian, lung and colorectal tumor samples, respectively (15). The expression of MELK was also upregulated in other human malignant tumors such as hepatocellular carcinoma (HCC) (16), gastric cancer (GC) (17), neuroblastoma (18, 19) and breast cancer (20–22). High MELK protein expression was significantly related with tumor number, tumor size and recurrence. Furthermore, elevated MELK protein expression was correlated with decreased overall survival and disease-free survival in various types of human cancer (9, 17–19). Notably, it revealed that high MELK protein expression was also significantly associated with higher pathological tumor-nodule-metastasis stage, vascular invasion (16). Moreover, the abnormal expression of MELK was related to cervical cancer metastasis at early stage (9). Speers and colleges identified MELK as a potential biomarker of radioresistance and target for radiosensitization in triple-negative breast cancer (TNBC) (20). These studies indicated that MELK may function as an oncogene in multiple types of cancer. Indeed, ectopic expression of MELK drastically promoted gastric cancer cell proliferation, migration and invasion *in vitro* and accelerated tumor growth and peritoneal spreading and metastasis in nude mice (8). Additionally, MELK overexpression confers radioresistance in ER-positive breast cancer cells with low baseline MELK expression (20). In contrast, knockdown of MELK significantly suppressed tumor cell proliferation, colony formation, stemness, and tumorigenicity, and induced apoptosis, mitosis, and DNA damage both *in vitro* and in nude mice models in gastric cancer (8), hepatocellular carcinoma (21) and cervical cancer (9). Li et al. found that targeting MELK by specific molecule inhibitor drastically diminished gastric cancer cell growth in preclinical GC patient-derived xenograft (PDX) mouse models (14, 17). In addition, inhibition of MELK resulted in suppression of migration, invasion and metastasis in gastric cancer (8, 17). Furthermore, in human TNBC, genetical or pharmacological inhibition of MELK induces radiation sensitivity *in vitro* and significantly delays xenograft tumor growth in combination with radiation therapy in multiple models (20). Therefore, the above studies suggest that MELK may be a predicting marker of poor prognosis or therapeutic target for human malignant tumors. However, up to now, the function of MELK in the development and progression of ESCC and its underlying molecular mechanisms remain unexplored.

In the current study, we detected MELK expression at mRNA and protein levels in cell lines and clinical specimens of ESCC, and determined the connection between MELK expression and metastasis in ESCC. By gain- and loss-of function, we explored the biological function of MELK in cell growth, migration, invasion and metastasis, and elucidated the possible underlying mechanisms *in vitro* and in animal models.

## Materials and Methods

### Cell Culture

Human ESCC cell lines TE-1, EC109, KYSE70, KYSE30, KYSE450, KYSE150, and EC9706 and one immortalized normal esophageal epithelial cell line Het-1A were obtained and cultured as our previously described (23). All cells were maintained in a humidified atmosphere (5% CO_2_) at 37°C and were recently tested for STR profiling and mycoplasma contamination.

### Human Tissue Specimens

A total 63 pairs of paraffin-embedded ESCC tissues (41 cases of primary and 22 cases of metastasis) used in this study were obtained from January 2015 to November 2018 in the First Affiliated Hospital of Henan University. Moreover, fresh tissues from 18 ESCC patients were collected and used for Western blotting analyses. None of the patients enrolled in the research received radiation or chemotherapy treatment prior to surgery. All patients signed the written informed consent documents prior to enrollment in the study, and the use of human tissues was approved by the Ethics Committee of the First Affiliated Hospital of Henan University.

### Quantitative Real-Time PCR (qRT-PCR)

qRT-PCR was performed as our previously described by using an Applied Biosystems 7900HT sequence detection system (Applied Biosystems) and SYBR Premix Ex Taq II (TaKaRa, Dalian, China) (23). PCR was conducted in a 20-μL volume reaction system containing 20 ng cDNA, 0.4 μmol/L paired primers and 10 μL SYBR Premix Ex Taq II according to the manufacture's manual. Relative expression differences were calculated with GAPDH by using the 2^−ΔΔCt^ method. The primer sequences used in this study were listed as follows: GAPDH-F, 5′-GAAGGTGAAGGTCGGAGTC-3′ and R, 5′-GAAGATGGTGATGGGATTTC-3′; MELK-F, 5′-CATTAGCCCTGAGAGGCGGTGC-3′ and R, 5′-GCCCGTCTCTGGCAGAACCCTT-3′. GAPDH was used as internal control.

### Cell Viability Assay

Cell viability was determined by 3-(4, 5-dimethyl-2-thiazolyl)-2, 5-diphenyl-2-H-tetrazolium bromide (MTT) assay according to the manufacturer's instruction (24). Briefly, cells (1,000 per well) were seeded in 96-well plates and incubated for 1 d, 2 d, 3 d, 4 d and 5 d. Twenty μL of MTT solution (5 mg/ml) was added to each well and the plates were maintained at 37 °C for another 4 h. The formed formazan crystals in each well were dissolved in 100 μl of DMSO. The Absorbance values was measured at a wavelength of 570 nm with a spectrophotometric plate reader (Synergy HT; BioTek, Winooski, VT).

### Focus Formation Assay

ESCC cells were plated in six-well plates (500/well) and cultured for 14 days. Colonies were fixed and stained with 0.1% crystal violet in 20% methanol for 20 min. Microscopic colonies composing more than 50 cells were counted (25).

### Soft Agar Assay

Soft agar assay was conducted as described in our previous study (26). Briefly, two thousand ESCC cells mixed with 0.4% bacto-agar (Sigma-Aldrich, Shanghai, China) medium containing 10% FBS were plated on a bottom layer of solidified 0.8% agar in 24-well plates (Corning). After incubation in a humidified incubator at 37° C for 2 weeks, the surviving colonies containing ≥50 cells were determined by microscope counting.

### Western Blotting Analysis

Western blotting analysis was performed using the whole cell lysates prepared in RIPA buffer containing 1 × protease inhibitor cocktail (Roche, Indianapolis, IN), 1 mM phenylmethylsulfonyl fluoride, 10 mM β-glycerophosphate and 10 mM NaF (26). The concentration of total protein was quantified by using Pierce^™^ BCA Protein Assay Kit (Thermo Fisher Scientific, #23250). Thirty ug of total proteins were separated by using 10–15% SDS-PAGE, and then transferred onto nitrocellulose membranes. After incubation with TBST buffer containing 5% dried skimmed milk for 1 h at room temperature, the membranes were hybridized with the indicated primary antibodies overnight. Finally, membranes were incubated with the corresponding HRP-conjugated secondary antibodies. Protein bands were detected using enhanced chemiluminescence (ECL) detection reagent (Beyotime, Shanghai, China) on a FluorChem M system (Protein Simple, USA). Antibodies used in this study are as follows: Antibodies against p-FOXM1 (Thr600, Cat. #14655), FOXM1 (Cat. #20459), phospho-SQSTM1 (Thr269/Ser272, Cat. #13121), SQSTM1 (Cat. #88588), phospho-eIF4B (Ser406, Cat. #5399) and eIF4B (Cat. #13088), S-phase kinase-associated protein 2 (SKP2) (Cat. #2652), Cyclin B1 (Cat. #4183), PLK1 (Cat. #4513), Aurora B (Cat. #3094), MMP-9 (Cat. #3852) and MMP-2 (Cat. #4022) were obtained from Cell Signaling Technology (Beverly, MA). Primary antibodies against MELK (Cat. #HPA017214) and Actin (clone AC-40, Cat. #A4700) were purchased from Sigma-Aldrich (Shanghai, China).

### Gelatin Zymography

The enzyme activity of MMP-2 and MMP-9 was measured by using gelatin zymography as previously described (27). Briefly, ESCC cells (1 × 10^5^) were plated in 12-well plates, Twenty-four hours later, the medium was changed with 500 μl serum-free medium, and continued to culture for 24 h. Tumor cell-conditioned medium (TCM) was collected and then stored at −80° C until used. The number of ESCC cells in each corresponding well was trypsinized and subsequently counted to allow appropriate correction of TCM loading for cell equivalents. To analyze the gelatinolytic activity of MMP-2 and MMP-9, aliquots of TCM were mixed with 4 × non-reducing sample buffer (250 mM Tris, 2.5% SDS, 12.5% glycerol, 0.1% bromophenol blue) and directly applied to a 10% SDS-PAGE gel containing 1% gelatin. After electrophoresis, the gel was soaked in 2.5% Triton X-100 to remove SDS and then incubated at 37°C overnight in the development buffer (50 mM Tris-HCl, pH 7.4, 0.2 M NaCl, 5 mM CaCl_2_, 0.02% NaN3), followed by staining with Coomassie Brilliant Blue R-250. After washing with decolorizing solution (20% methanol, 10% acetic acid), the gel was scanned with a FluorChem M system (Protein Simple, USA).

### Wound Healing Assay

ESCC cells were pretreated with 10 μg/mL mitomycin C (Selleck, Shanghai, China) for 2 h to inhibit cell proliferation (28). Cells were then collected and plated in 6-well plates (Corning) and grown to form a confluence cell monolayer for overnight. After scratching with a sterile pipette tip, the wells were washed with PBS to remove the floating cells. Cells were then cultured in serum-free medium and recorded at 0 h, 24 h, and 48 h post-scratching by using an inverted microscope. The percentage of wound closure was calculated by the following formula: wound closure (%) = (original gap distance—gap distance at the indicated time)/original gap distance × 100% as our previously described (23, 29).

### Transwell Assays

Transwell assays were performed by using transwell chambers (8 μm pore size, Corning) to examine the migration and invasion of ESCC cells as previously described (30). Briefly, tumor cells were pretreated with 10 μg/mL mitomycin C (Selleck, Shanghai, China) for 2 h. For the migration experiment, Approximately 5 × 10^4^ cells were placed into the upper chamber with 200 μl serum-free DMEM medium, The lower compartment filled with DMEM medium supplement with 20% FBS was used as the chemoattractant. For the invasion assay, the upper surface of chamber membrane were coated with 50 μL Matrigel (Cat. #354234, BD Biosciences, CA) to form a matrix barrier, and then 1 × 10^5^ tumor cells were added to the upper chamber. After incubation for 24 h in a humidified incubator containing 5% CO_2_ at 37°C, the cells on the lower surface of the chamber membrane were fixed with 4.0% paraformaldehyde prior to 0.1% crystal violet staining. Pictures were then captured under a light microscope at 10 × and the mean number of migrated or invaded cells were counted in five random fields.

### Transfection of Short Hairpin RNA (shRNA) and Plasmids

Plasmids including pCMV-Flag-His-puro-MELK (MELK), pCMV-Flag-His-puro-FOXM1 (FOXM1) and the empty vector pCMV-Flag-His-puro (Vector) were purchased from Transheep (Shanghai, China). For MELK overexpression, cells were transfected with 2 μg plasmids (MELK or Vector) for 48 h by using the Lipofectamine 2000 reagent (Invitrogen, Thermo Fisher Scientific, Inc.) following the manufacturer's manual (23). Cells were then selected in the presence of 1.5 μg/mL puromycin (Sigma-Aldrich). After selection for 3 weeks, the stable colonies were picked up and expanded. For ectopic expression of FOXM1, the MELK-silenced KYSE30 and EC9706 cells were transfected with 2 μg plasmids (FOXM1 or Vector) for 48 h by using the Lipofectamine 2000 reagent. The cells were then exposed to puromycin (1.5 μg/mL) for 3 weeks.

Specific shRNAs targeting MELK (shMELK#1 and shMELK#2) or FOXM1 (shFOXM1#1 and shFOXM1#2) and a scramble shRNA (shNC) were obtained from Sigma-Aldrich. The indicated sequences were described in Table S1. Lentiviruses production and subsequently tranfection were performed as our previously described (23). The cells were then cultured with puromycin for 3 weeks to establish stable cells.

### Mouse Models of Tumorigenesis and Metastasis

Male BALB/c nude mice (5 week-old, 18–20 g) were purchased from Beijing Vital River Laboratory Animal Technology Co (Beijing, China). All mice were bred and maintained in barrier system at the animal facility of Henan University with controlled humidity (40–50%), temperature (20 ± 2 °C) and a lighting cycle of 12 h light/darkness. The standard pellet food and water were provided *ad libitum* during the experimental period. All the animal studies were approved by the Henan University Institutional Animal Care and Use Committee.

For tumorigenic model, mice (6 mice per group) were subcutaneously implanted with the indicated ESCC cells with MELK overexpression or knockdown (5 × 10^6^ per mice) into the left dorsal flank of each mouse (26). The formed tumors were measured with calipers every other day and calculated by the formula as follows: tumor volume (mm^3^) = (W^2^ × L)/0.5, where *W* represents the smallest diameter and *L* is the diameter perpendicular to *W*. All mice were anesthetized with isoflurane before killed by cervical dislocation after injection for about 3 weeks. Tumors were immediately removed, weighed, fixed in formalin or kept at −80 °C.

For the experimental metastasis assay, Approximately 1 × 10^6^ ESCC cells were intravenously injected into the nude mice (6 mice/group) via the lateral tail vein (31). After injection for 8 weeks, all of the mice were killed by cervical dislocation, the lungs were dissected and fixed in Bouin's solution as previously described (26). Twenty-four hours later, metastatic colonies on the surface of the lungs in each group were counted, and the tumor lesions within the lungs was further confirmed by hematoxylin and eosin (H&E) staining.

### Immunohistochemistry (IHC) Staining

The procedure of IHC staining was performed as described in our previous studies (29). Briefly, After deparaffinization and rehydration, the tissue slides were treated with 3% H_2_O_2_ for 10 min to exhaust the endogenous peroxidase, and the antigens were retrieved with a microwave oven for 15 min in the presence of 10 mM citrate solution (pH 6.0). After blocking in 5% bovine serum albumin for 20 min, the slides were incubated with the indicated primary antibodies in a humidified container at 4°C for 12 h. Sides were developed with the reagents of EnVision + System-HRP (DAB; DAKO) according to manufacturer's protocol, and then counterstained with hematoxylin. Represent images were captured by using a phase contrast microscope (Leica, Germany).

For human ESCC samples, IHC scoring was carried out as our previously described (29). The intensity score was defined according to the four grades as follows: 3, strong staining; 2, moderate staining; 1, weak staining; 0, no staining. The percentage of positively stained cells was scored as 0–100%. The percentage score multiplied by the intensity scores to obtain the overall score (0–3), which represented the expression of MELK protein.

### Statistical Analysis

All experiments were carried out for at least five independent experiments, and results were presented as mean ± standard deviation (SD). Statistical comparisons between two groups were performed with the used of Student's *t*-test; Three or more groups were compared by using a one-way ANOVA with Tukey's *post hoc* test. The value of *P* < 0.05 (two-sided) was considered as statistically significant. GraphPad Prism software version 7.0 (San Diego, CA) was conducted for all the statistical analyses.

## Results

### MELK Is Highly Expressed in ESCC

**Figure 1:**
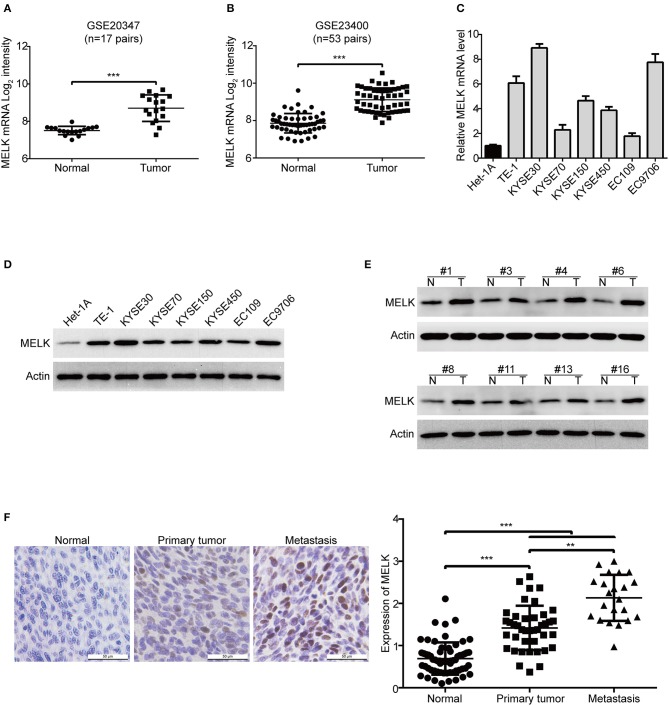
MELK is highly expressed in ESCC. **(A,B)** MELK mRNA expression was calculated from two Gene Expression Omnibus (GEO) cancer datasets, GSE20347 (*n* = 17 pairs) **(A)** and GSE23400 (*n* = 53 pairs) **(B)**, both of which were examined cDNA microarray from primary ESCC and the corresponding normal tissues. The levels of MELK in one immortalized esophageal epithelial cell line Het-1A and several ESCC cell lines were determined by qRT-PCR **(C)** and immunoblotting analysis **(D)**. **(E)** Western blotting analysis was used to determine MELK expression in 18 pairs of ESCC tumor and matched normal tissues. **(F)** Representative IHC micrographs (left) and summary bar chart (right) of MELK protein expression in 63 pairs of ESCC tissues (all ESCC cases had paired normal tissues; 41 cases of primary and 22 cases of metastasis). Scale bars, 50 μm. **P < 0.01 and ***P < 0.001 by one-way ANOVA, *post-hoc* intergroup comparisons, Tukey's text.

To determine MELK expression in human ESCC, we firstly analyzed the transcript expression of MELK in two cohorts of ESCC patients from GEO database, the results showed that the mRNA level of MELK in GSE20347 dataset (32) was much higher in ESCC tissues than that in the corresponding normal tissues (Figure 1A); Similar results were observed in GSE23400 dataset (33), which composes of 53 pairs of ESCC specimens (tumor and matched normal cases) (Figure 1B). Next, we detected the expression of MELK in ESCC cell lines by using qRT-PCR and western blotting analysis. As illustrated in Figure 1C, the mRNA expression of MELK was elevated in all tested ESCC cells, compared to that in immortalized esophageal epithelial Het-1A cells. Consistently, the levels of MELK protein were obviously higher in ESCC cells, especially in TE-1, KYSE30, KYSE450 and EC9706 cells (Figure 1D). We also determined MELK protein in 18 pairs of ESCC tumors and the corresponding normal tissues by using immunoblotting analysis. The data displayed that the expression of MELK protein was elevated in all tested ESCC tissues, compared to the matched controls (Figure 1E). In addition, we also detected the protein level of MELK in 63 pairs of human ESCC and the matched normal specimens by using IHC staining. As illustrated in Figure 1F, compared to that in the adjacent normal esophageal tissues, the expression of MELK was markedly upregulated in ESCC tissues. Interestingly, the levels of MELK protein in the metastatic ESCC tissues were much higher than those in primary tissues. The above data demonstrated that MELK is highly expressed in ESCC and might play a critical role in tumorigenicity and metastasis. Given that the expression of MELK was lower in KYSE70 and EC109 cells, they were used for gain-of-function analysis. In contrast, KYSE30 and EC9706 cells with higher expression of MELK were used for loss-of-function experiments.

### MELK Promotes the Growth ESCC Cells

**Figure 2:**
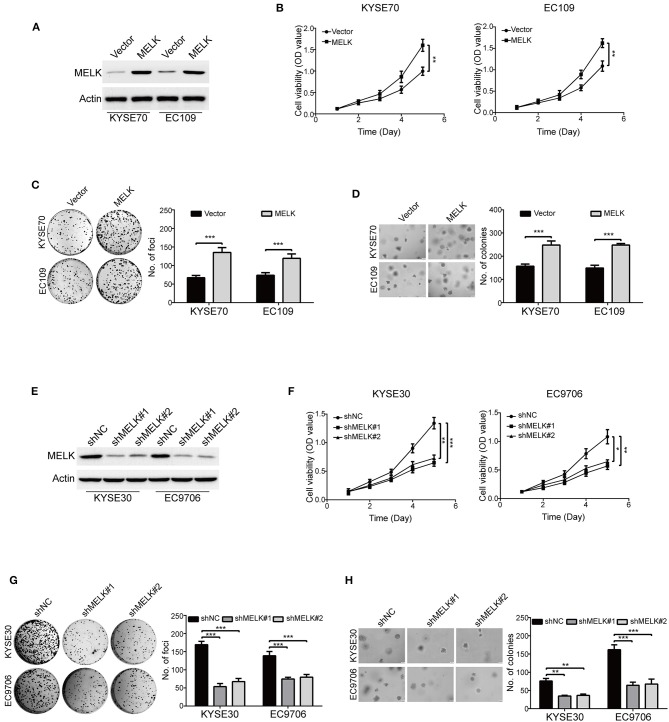
MELK accelerates ESCC cell growth. **(A)** Western blotting analysis of MELK expression in ESCC cells (KYSE70 and EC109) stably transfected with MELK plasmid (MELK) or the empty vector (Vector). **(B)** MTT assay was used to measure the viabilities of cells with MELK overexpression (*n* = 5). Representative images (left) and statistics (right) of focus formation assay (*n* = 6) **(C)** and colony formation in soft agar assay (*n* = 6) **(D)** in ESCC cells stably transfected with MELK or empty vector. Scale bars, 100 μm. **P < 0.01 and ***P < 0.001 by Student's *t*-test. **(E)** The effect of MELK-targeting shRNAs was confirmed by immunoblotting analysis. KYSE30 and EC9706 cells were stably transfected with two shRNAs (shMELK#1 and shMELK#2) against MELK or the scrambled shRNA (shNC). **(F)** The viabilities of ESCC cells with stable MELK depletion was measured by MTT assay (*n* = 5). Silencing MELK could significantly decrease the number of focus formation (*n* = 6 per group) **(G)** and colony formation in soft agar (*n* = 6 per group) **(H)**. Quantification of colonies were shown in the bar chart. Scale bars, 200 μm. *P < 0.05, **P < 0.01, ***P < 0.001 vs. shNC; *P*-values were obtained by one-way ANOVA with *post-hoc* intergroup comparison with the Tukey's test.

To examine the oncogene function of MELK in ESCC, a MELK plasmid was stably transduced into KYSE70 and EC109 cells (Figure 2A). MTT assay showed that the cell viabilities were greatly increased by MELK in both KYSE70 and EC109 cells, compared with their corresponding control (Figure 2B). In addition, the focus formation assay in solid plates indicated that ectopic expression of MELK significantly promoted the colony formation of the tested cell lines of ESCC (Figure 2C). Furthermore, we also performed soft agar assay to determine the effect of MELK on ESCC cell growth. As shown in Figure 2D, the number of individual colonies was much larger in MELK-expressing KYSE70 cells than that of KYSE70-Vector group; Similar phenomenon was found in EC109 cells (Figure 2D, right). We next explored whether silencing MELK suppresses *in vitro* tumorigenicity of ESCC cells. KYSE30 and EC9706 cells were stably transduced by using lentiviral constructs with two different shRNA oligonucleotides targeting the coding sequence of human MELK (shMELK#1 and shMELK#2). The silencing efficiency was confirmed by immunoblotting analysis, and the results showed that the expression of MELK was specifically downregulated by both shRNAs (Figure 2E). Interestingly, knockdown of MELK drastically inhibited the cell viabilities in both KYSE30 and EC9706 cells (Figure 2F). In addition, silencing MELK also effectively attenuated the colony formation abilities of ESCC cells in both solid plates and soft agar assays, compared to those cells transfected with shNC (Figures 2G,H). Collectively, these data confirmed that MELK enhanced the growth of ESCC cells *in vitro*.

### MELK Enhances ESCC Cell Migration and Invasion

**Figure 3:**
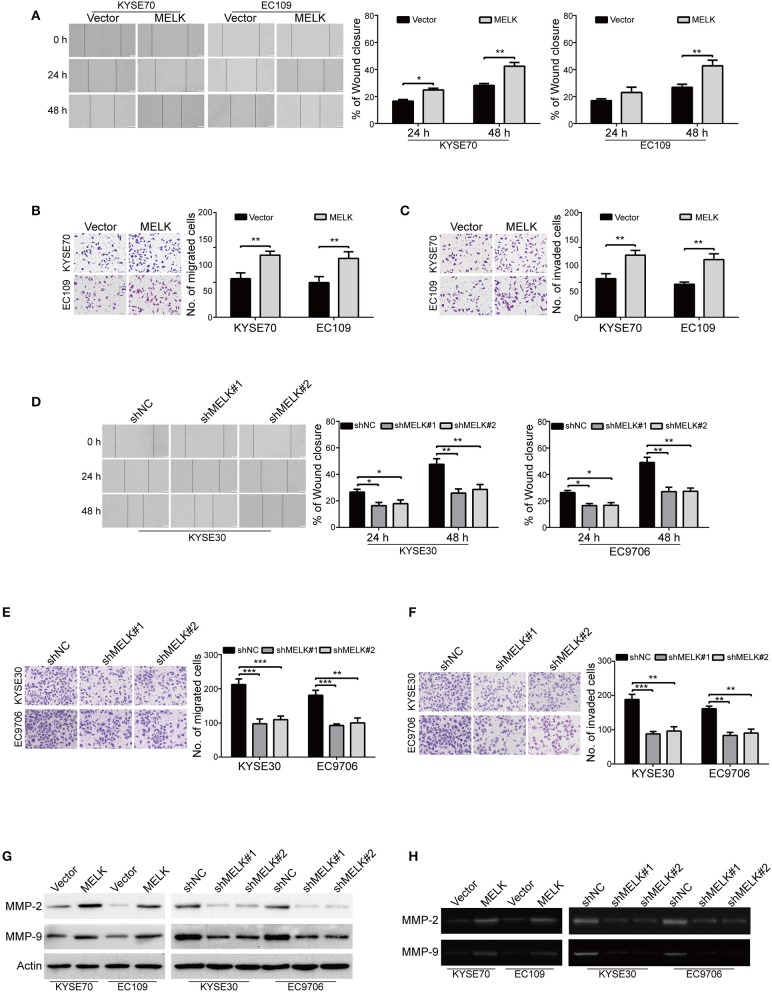
MELK promotes the migration and invasion of ESCC cells. **(A)** Wound-healing assay was conducted to determine the effect of MELK on ESCC cell migration. Column and error bar represents mean ± SD (*n* = 5 per group, right). **(B,C)** Representative images (left) and summary bar chart (right) of cells that migrated through a membrane **(B)** or invaded through a Matrigel-coated membrane **(C)** *n* = 5, Scale bars: 100 μm. *P < 0.05 and **P < 0.01 vs. Vector group; *P*-values wereobtained by Student's *t*-test. **(D)** knockdown of MELK dramatically reduced the migratory ability of ESCC cells in a wound-healing assay. **(E,F)** Transwell analysis was performed to detect the migratory **(E)** and invasive **(F)** abilities of ESCC cells with MELK deletion. Representative images (left) and summary bar chart (right) are shown. Column and error bar represents mean ± SD (*n* = 5). *P < 0.05, **P < 0.01, ***P < 0.001 compared with shNC, one-way ANOVA, *post-hoc* intergroup comparisons, Tukey's text. **(G)** The protein levels of MMP-2 and MMP-9 were measured by immunoblotting assay in ESCC cells with MELK overexpression (left) or knockdown (right). **(H)** Analysis of MMP-2 and MMP-9 activity in TCM by gelatin zymography. TCM was collected from tumor cells with MELK overexpression (left) or knockdown (right).

We then explored whether MELK contributes to enhancing the abilities of ESCC cell migration and invasion. As shown in Figure 3A, overexpression of MELK drastically promoted the migration of ESCC cells at 24 h, compared to that of the control cells transduced with Vector, this phenomenon was more obvious at 48 h. To further demonstrate these findings, transwell assay was conducted to examine the effects of MELK on ESCC cell migration. As illustrated in Figure 3B, the number of tumor cells that migrated through the membrane of transwell chamber was significantly increased by MELK in both KYSE70 and EC109 cells. We then investigated whether ectopic expression of MELK promotes ESCC cell invasion. As shown in Figure 3C, overexpression of MELK remarkably increased the number of invaded cells in both tested cell lines of ESCC. Next, We determined whether knockdown of MELK attenuates the abilities of ESCC cell migration and invasion *in vitro*. The data showed that the migration abilities of KYSE30 and EC9706 cells were obviously inhibited by MELK shRNAs in a wound healing assay (Figure 3D). Besides, transwell assay also showed that knocking down MELK by shRNAs drastically diminished the numbers of migration cells (Figure 3E). Consistently, the number of tumor cells invaded through Matrigel were decreased in MELK-depleted KYSE30 cells, compared to that of the control group with shNC transfection (Figure 3F); A similar phenomenon was also observed in EC9706 cells (Figure 3F). Additionally, immunoblotting analysis showed that the levels of MMP-2 and MMP-9 protein were markedly unregulated in MELK-expressing KYSE70 and EC109 cells, but were drastically reduced by MELK shRNAs in cell lysates derived from KYSE30 and EC9706 cells, compared to their corresponding controls (Figure 3G). Gelatin zymography also showed that, compared with TCM obtained from control cells, those from MELK-overexpressing KYSE70 and EC109 cells displayed a significant upregulation in the enzyme activity of MMP-2 and MMP-9 (Figure 3H), whereas TCM from MELK-depleted KYSE30 and EC9706 cells revealed an obvious reduction in MMP-2 and MMP-9 activity (Figure 3H).

Altogether, these data indicated that MELK conferred ESCC cell migration and invasion, probably via promoting the expression and activity of MMP-2 and MMP-9.

### MELK Promotes the Malignant Phenotypes of ESCC Cells Through Activation of FOXM1 Signaling Pathway

**Figure 4:**
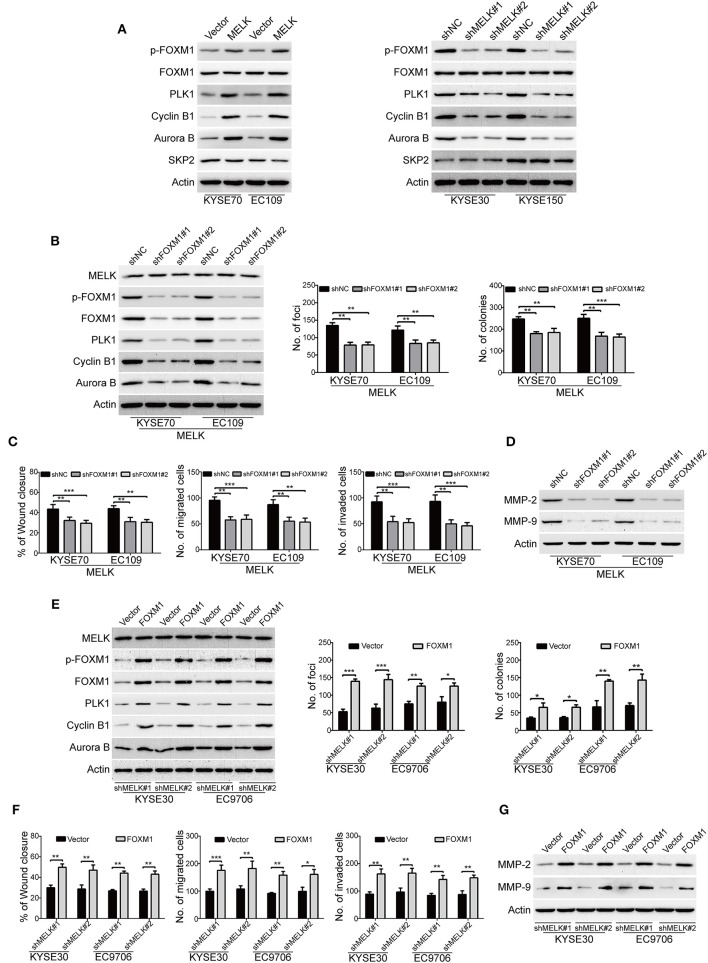
MELK promotes the growth, migration and invasion through activation of FOXM1 signaling. **(A)** Immunoblotting analysis was used to determine the protein levels of p-FOXM1 (Thr600), FOXM1, PLK1, Cyclin B1, Aurora B and SKP2 in ESCC cells with MELK-overexpression (KYSE70 and EC109) or knockdown (KYSE30 and EC9706). Actin served as an internal control. **(B)** Western blotting analysis was carried out to measure the efficiency of FOXM1 shRNAs (shFOXM1#1 and shFOXM1#2) in MELK-depleted ESCC cells (left). Knockdown of FOXM1 drastically inhibited the frequency of focus formation (middle) and colony formation in soft agar (right). Column and error bar represents mean ± SD (*n* = 6 per group). **P < 0.01, ***P < 0.001, *P*-values were obtained by one-way ANOVA, *post-hoc* intergroup comparisons, Tukey's text. **(C)** Knocking down FOXM1 significantly attenuated the migratory ability of MELK-overexpressing KYSE70 and EC109 cells in a wound-healing assay (left). Downregulation of FOXM1 reduced the migratory (middle) and invasive abilities (right) of ESCC cells with MELK overexpression. Column and error bar represents mean ± SD (*n* = 5). **P < 0.01, ***P < 0.001, one-way ANOVA with *post-hoc* intergroup comparison with the Tukey's test. **(D)** Silencing FOXM1 attenuated the protein levels of MMP-2 and MMP-9 in MELK-overexpressing ESCC cells. **(E)** Western blotting analysis was used to detect the expression of FOXM1 in shMELK-depleted KYSE30 and EC9706 cells after transfection of FOXM1 plasmid or empty vector. Ectopic expression of FOXM1 promoted the number of focus formation (middle) and colony formation in soft agar (right). Column and error bar represents mean ± SD (*n* = 6 per group). **(F)** Enforced expression of FOXM1 enhanced the migratory ability of shMELK-silenced ESCC cells in a wound-healing assay (left). Ectopic expression of FOXM1 rescued the migration (middle) and invasion (right) of shMELK-depleted ESCC cells. Column and error bar represents mean ± SD (*n* = 5 per group). *P < 0.05, **P < 0.01 and ***P < 0.001 by Student's *t*-test. **(G)** Overexpression of FOXM1 restored MMP-2 and MMP-9 expression in shMELK-silenced KYSE30 and EC9706 cells.

We subsequently investigated the underlying molecular mechanisms of MELK involved in the malignant phenotypes of ESCC cells. Previous studies demonstrated that MELK interacts with and phosphorylates its downstream substrates including FOXM1, eukaryotic translation initiation factor 4B (eIF4B) and SQSTM1, and thus promotes the malignant phenotype of human cancer (34–36). We then investigated whether MELK affects the phosphorylation of these candidate substrates in ESCC cells. Intriguingly, immunoblotting analysis showed that overexpression of MELK obviously promoted the expression of phospho-FOXM1 (Thr600) in both KYSE70 and EC109 cells (Figure 4A, left). In contrast, knockdown of MELK by both specific shRNAs drastically inhibited the phosphorylation of FOXM1 (Thr600) (Figure 4A, right); But, overexpression or knockdown of MELK hardly influenced the protein levels of FOXM1 (Figure 4A), phospho-eIF4B (Ser406), phospho-SQSTM1 (Thr269/Ser272), SQSTM1 and eIF4B (Figure S1). These data suggested that MELK might promoted ESCC cell growth, migration and invasion via phosphorylation and activation of FOXM1 signaling pathway. To further identify these results, we also detected its downstream targets including PLK1, Cyclin B1, Aurora B and SKP2 by using Western blotting analysis. Interestingly, ectopic expression of MELK drastically upregulated the protein levels of PLK1, Cyclin B1 and Aurora B, but not SKP2 (Figure 4A, left); Vice versa, silencing MELK led to an opposing results (Figure 4A, right). To determine whether knockdown of FOXM1 attenuates the oncogene function of MELK in ESCC cells, two different shRNA oligonucleotides targeting the coding sequence of human FOXM1 (shFOXM1#1 and shFOXM1#2) were stably transduced into MELK-overexpressing KYSE70 and EC109 cells. The efficiency of FOXM1 shRNAs was measured by immunoblotting analysis, and the data indicated that FOXM1 expression was specifically downregulated by both shRNAs (Figure 4B, left). As expected, silencing FOXM1 drastically inhibited the protein levels of p-FOXM1 (T600), FOXM1, PLK1, Cyclin B1, Aurora B, but not MELK in both tested cell lines with MELK overexpression (Figure 4B, left). Consistently, knockdown of FOXM1 in KYSE70-MELK and EC109-MELK cells obviously decreased the number of colonies in solid plates (Figure 4B, middle) and attenuated the colony formation abilities in soft agar assays (Figure 4B, right). Moreover, the wound-healing and Boyden chamber assays displayed that the migration and invasion of MELK-overexpressing KYSE70 and EC109 cells were obviously inhibited by FOXM1 shRNAs (Figure 4C). Furthermore, knocking down of FOXM1 drastically suppressed MMP-2 and MMP-9 expression in MELK-overexpressing ESCC cells, compared to the corresponding control group (Figure 4D).

Next, we investigated whether ectopic expression of FOXM1 rescued the loss of MELK function in ESCC cells. FOXM1-overexpressing plasmid was stably transduced into MELK-depleted KYSE30 and EC9706 cells, and then the expression of FOXM1 was detected by Western blotting (Figure 4E, left panel). As shown in Figure 4E, the protein levels of p-FOXM1 (T600), FOXM1 and its downstream targets including PLK1, Cyclin B1, Aurora B were drastically upregulated by FOXM1 in both tested cell lines with MELK depletion. Enforced expression of FOXM1 in MELK-silenced KYSE30 and EC9706 cells also attenuated the inhibitory effects on the colony formation abilities in both solid plates and soft agar assays (Figure 4E, middle and right panels). Additionally, the data from wound-healing and Boyden chamber assays indicated that the migration and invasion were obviously rescued by FOXM1 in KYSE30 and EC9706 cells with MELK downregulation (Figure 4F). Immunoblotting analysis also showed that ectopic expression of FOXM1 remarkably restored MMP-2 and MMP-9 expression in MELK-silenced ESCC cells (Figure 4G).

The above data suggested that FOXM1 is required for the function of MELK in ESCC cells.

### MELK Enhances Tumor Growth in ESCC

**Figure 5:**
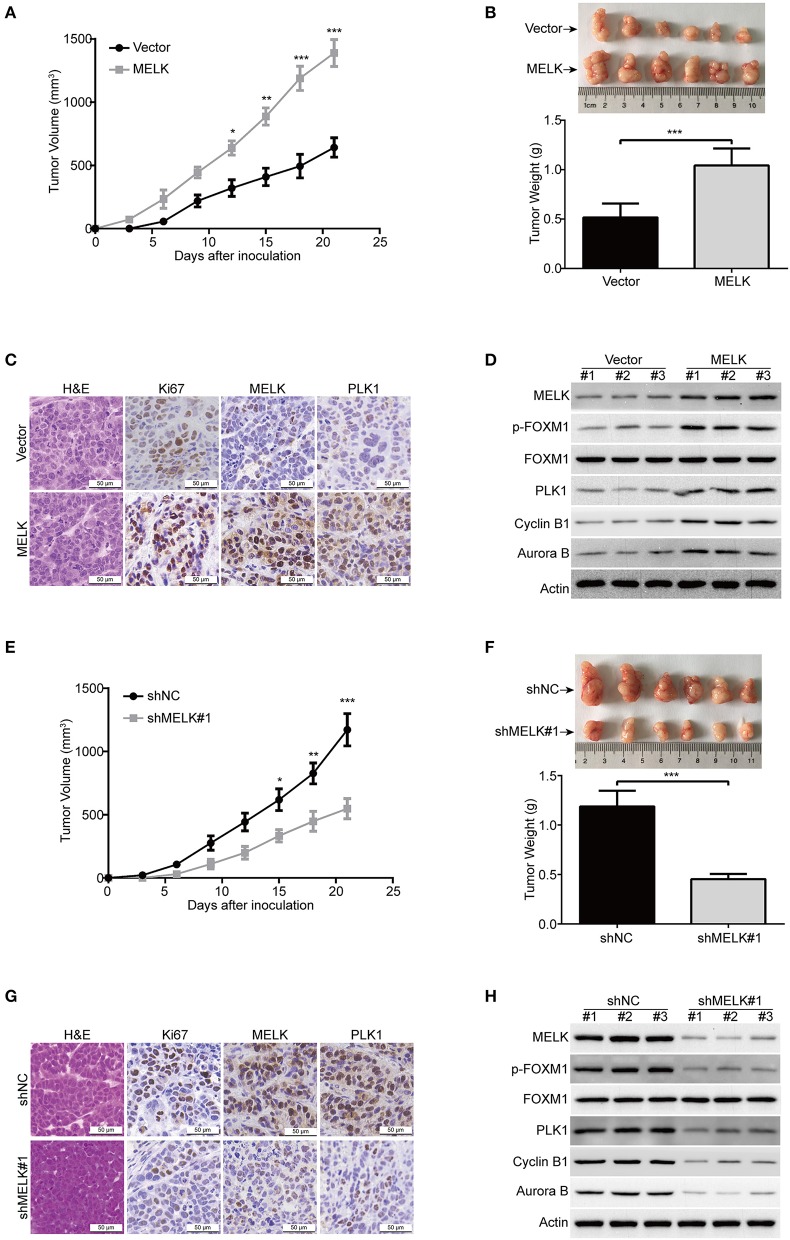
MELK promotes ESCC cell growth in nude mice. **(A)** The growth curves of subcutaneous xenografts of MELK-overexpressing EC109 cells are shown. Data represents mean ± SD. **(B)** Upper, representative tumors derived from EC109 cells with or without MELK overexpression are presented; Lower, comparison of tumor weights in control (Vector) and MELK-overexpressing group (*n* = 6). **(C)** Immunohistochemical analysis of Ki67, MELK, and PLK1 in xenograft tissues from mice. H&E-stained tissues of the same xenografts are shown. **(D)** Immunoblotting analysis was used to detect the protein levels of MELK, p-FOXM1 (Thr600), FOXM1, PLK1, Cyclin B1 and Aurora B proteins in xenograft tissues. **(E)** The growth curves of subcutaneous xenografts of KYSE30-shNC and KYSE30-shMELK cells are shown. **(F)** Top, representative tumors from the control and experiment group are shown; bottom, comparison of tumor weights in control (shNC) and MELK-depleted group (*n* = 6 per group). **(G)** IHC staining showing the expression of MELK, PLK1 and Ki67 in xenograft tissues from mice. H&E-stained tumor tissues of the same xenografts are presented. **(H)** Immunoblotting analysis was used to detect the protein levels of MELK, p-FOXM1 (Thr600), FOXM1, PLK1, Cyclin B1 and Aurora B proteins in xenograft tissues derived from control (shNC) and MELK-deleted group. Scale bars: 50 μm. *P < 0.05, **P < 0.01, ***P < 0.001 by Student's *t*-test.

Considering that MELK promoted cellular growth and colony formation of KYSE70 and EC109 cells, we next determine whether MELK promotes the tumorigenisis of ESCC cells in nude mice. The tumor growth curve of EC109-MELK group was much higher than that of the EC109-Vector group (Figure 5A), suggesting that MELK promoted the tumor growth of ESCC cells *in vivo*. Consistent with these data, the size and weight of tumor in MELK-overexpressing mice were obviously increased, compared with those of the corresponding control mice (Figure 5B). Additionally, IHC staining showed that Ki67 expression (a proliferation marker) was remarkably enhanced by MELK (Figure 5C). Moreover, the expression of MELK and PLK1 was greatly upregulated in MELK-overexpressing group, compared to that derived from the Vector-treated mice (Figure 5C). Furthermore, immunoblotting assay also showed that the protein levels of MELK, phospho-FOXM1 (T600), and its downstream targets (PLK1, Cyclin B1 and Aurora B) were much higher in tumor cell lysates derived from MELK-overexpressing mice than those from the control group (Figure 5D). We then determined whether knockdown of MELK inhibits ESCC cell growth in animal models using KYSE30 cells. As illustrated in Figure 5E, the growth rate of solid tumors in MELK-depleted mice was slower, compared with that of the control group. Similarly, the size and weight of tumors were also obviously inhibited by MELK shRNA (Figure 5F), suggesting that silencing MELK suppressed the tumor growth of ESCC cell in nude mice. Consistently, IHC staining displayed that the levels of Ki67, MELK, and PLK1 were significantly impaired by MELK shRNA (Figure 5G). In addition, immunoblotting analysis also displayed that the protein levels of MELK, p-FOXM1 (T600), PLK1, Cyclin B1 and Aurora B were drastically diminished in cell lysates derived from MELK-depleted mice, compared to those from the shNC group (Figure 5H).

### MELK Promotes ESCC Cell Metastasis

**Figure 6:**
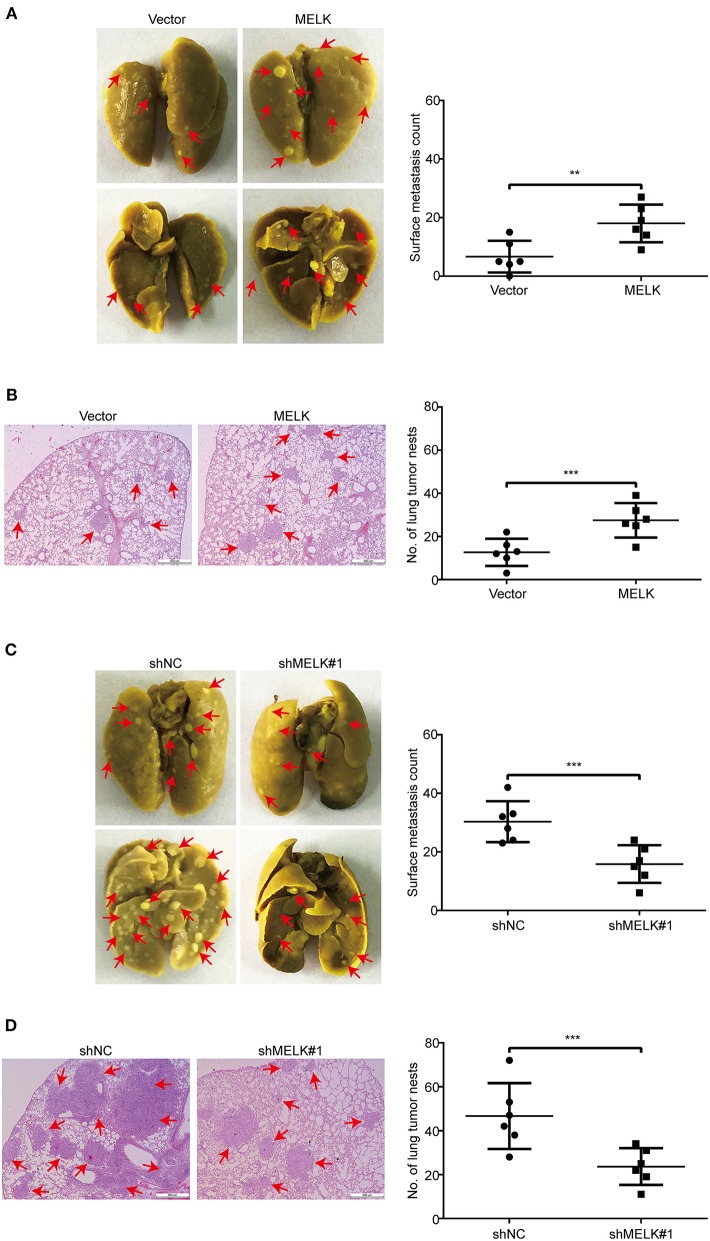
MELK enhances ESCC cell metastasis in nude mice. **(A)** EC109 cells with or without MELK overexpression were intravenously injected into nude mice (*n* = 6 per group) through the tail vein. (Left) Representative images of excised lungs after 8 weeks injection are shown (arrows indicate the metastatic foci). (Right) Graph showing the number of surface metastatic nodules in the lungs. **(B)** Lung metastases in each mice were stained with H&E. Arrows indicate the metastatic colonization of tumor cells in the lung tissues (left). The number of lung tumor nests in each group was counted (right). **(C)** Effects of MELK depletion on the location of KYSE30 cells in the lung of nude mice after injection of cells via tail-vein at 8 weeks. (Left) Representative images of excised lungs. (Right) Graph showing the number of surface metastases in the lung tissues. **(D)** Lung metastases in each mice were confirmed by H&E staining. Arrow indicates the metastatic colonization of tumor cells in the lung tissues (left). (Right) The number of lung tumor nests in each group was counted and presented by mean ± SD. Scale bars, 500 μm. **P < 0.01 and ***P < 0.001 by Student's *t*-test.

Given that ectopic expression of MELK significantly enhanced the abilities of migration and invasion of KYSE70 and EC109 cells *in vitro*, we next investigate whether MELK promotes the lung metastasis of tumor cells in nude mice. EC109 cells with or without MELK overexpression were intravenously injected into the nude mice. Eight weeks later, the lung tissues derived from mice were dissected and fixed in Bouin's solution. As shown in Figure 6A, the average numbers of metastatic foci in the lungs were 12.7 and 27.5 in control and MELK-overexpressing groups, respectively. Likewise, H&E staining also showed that the number and size of metastatic tumors were significantly upregulated in the lungs of MELK-overexpressing mice (Figure 6B). These results indicated that MELK significantly promoted ESCC cell metastasis *in vivo*. To further confirm this conclusion, we then examined whether silencing MELK suppresses ESCC cell metastasis in nude mice. KYSE30-shNC and KYSE30-shMELK#1 cells were intravenously injected into the nude mice. As illustrated in Figure 6C, the number of metastatic tumor nodules in the lungs was much smaller in MELK-downregulated group than that of the control group. Likewise, H&E staining also showed that the number and size of metastatic tumors were significantly decreased in the lungs derived from MELK-depleted mice, compared to those from the shNC group (Figure 6D). Taken together, these data indicate that MELK functions as a pro-metastatic factor in ESCC *in vivo*.

## Discussion

In the present work, we discovered that MELK was overexpressed in clinical ESCC samples, especially in those with metastasis, suggesting that MELK might function as an oncogene in ESCC. As expected, overexpression of MELK enhanced the abilities of ESCC cell proliferation, colony formation, migration and invasion. More importantly, enforced expression of MELK also promoted tumor growth and metastasis in animal models. Vice versa, knocking down MELK led to an almost completely opposing effect. Mechanistic analysis revealed that aberrant activation of FOXM1 signaling pathway might be one crucial mechanism for MELK to induce the aggressive malignant phenotypes of ESCC cells. To the best of our current knowledge, we are the first to detect the expression of MELK, and to elucidate its biological role and underlying mechanism in ESCC cell growth and metastasis, using *in vitro* and xenograft models in nude mice.

Accumulating evidence has shown that MELK was highly expressed in various kinds of human cancer and this expression was correlated with the development and progression of malignancy tumors (9, 19). In this study, we discovered that MELK was upregulated in all tested seven ESCC cell lines at both mRNA and protein levels. In addition, we also found that MELK was aberrantly expressed in clinical specimens of ESCC patients. Consistent with our findings, Janostiak et al. found that MELK was highly overexpressed in melanoma and played a critical role in promoting melanoma growth (36). Besides, MELK is also abundantly expressed in glioblastoma multiforme (GBM) and is essential for the proliferation of cancer cells and cancer stem cells (34). Consistently, we also confirmed that enforced expression of MELK in ESCC cells by transfection with MELK-overexpressing plasmid significantly promoted the tumor cell proliferation, colony formation, anchorage-independent growth *in vitro*, and also enhanced the tumor growth in nude mice models. More importantly, knocking down of MELK by lentiviral shRNA resulted in an opposite phenomenon. Considering that MELK knockout mice are viable and display no obvious adverse phenotypes (37), therefore, MELK might be a potential druggable target for ESCC treatment. However, a few reports have demonstrated that MELK was not required for cell division in basal-like breast cancer, melanoma and colorectal cancer (38, 39). This findings suggest that MELK might play a role as oncogene or not, depending on the tumor type context.

FOXM1 is one of the most important members of the Forkhead box (FOX) superfamily that contains more than 50 members with a conserved winged-helix DNA binding domain (40, 41). As a transcription factor, FOXM1 crosstalk with multiple proteins including PLK1, Cyclin B1 and Aurora B, and thus plays a central role in tumor aggressiveness. Overexpression of FOXM1 has been found in a variety of human malignancies including ESCC (42–45). High expression levels of FOXM1 was positively correlated with large tumor size, poor differentiation degree, deep invasion, and low survival rate and might serve as an independent prognostic indicator for determining prognosis of ESCC patients (42, 43). Moreover, ectopic expression of FOXM1 drastically enhanced the capability of invasion and migration (45) and silencing of FOXM1 obviously inhibited the proliferation and migration in ESCC cells (46). In this study, we found that FOXM1 was a critical phosphorylation target of MELK. By activating FOXM1, MELK significantly promoted the malignant phenotypes of ESCC cells, this conclusion was based on the following facts: (1) Ectopic expression of MELK promoted, while lentiviral shRNA mediated gene silencing diminished the expression of p-FOXM1 (T600) and its downstream targets including Cyclin B1, PLK1, Aurora B and Survivin both *in vitro* and *in vivo*; (2) Overexpression of FOXM1 diminished the inhibitory effect of MELK shRNAs on ESCC cell growth, migration and invasion. In contrast, silencing FOXM1 by shRNAs dramatically attenuated the tumor-promoting effects of MELK on ESCC cells; (3) Overexpression or knockdown of FOXM1 hardly changed the protein levels of MELK. Consistent with our results, Joshi et al. found that MELK-driven FOXM1 phosphorylation plays a pivotal role in proliferation of glioma stem cells (34).

Tumor metastasis is a complex process in which the tumor cells move from a primary site to progressively colonize distant organs, and is one of the most important contributors to the deaths of patients with multiple types of malignant tumors including ESCC (47). Previous studies demonstrated that highly expressed MELK correlated with the histopathological grading and tumor metastasis in patients with cervical cancer (9). Overexpression of MELK was also associated with distant metastasis, lymph node involvement and poor prognosis in GC patients (17). In HCC, high MELK protein expression was not only related with short disease-free survival and overall survival but also correlated with vascular invasion and higher pathological tumor-nodule-metastasis stage (16). Consistently, we observed that the expression of MELK was much higher in metastatic ESCC tissues, compared to that from primary tumors. These studies suggested that MELK might played a crucial role in tumor metastasis. In addition, we also confirmed that overexpression of MELK greatly promoted the capacity of migration and invasion, and upregulated the expression and enzyme activity of MMP-2 and MMP-9 in all two tested ESCC cell lines. More importantly, ectopic expression of MELK also enhanced the lung metastasis of EC109 cells in animal model. Vice versa, the migration, invasion and metastasis of ESCC cells were remarkably inhibited after knocking down of MELK by lentiviral shRNA. In line with our findings, ectopic expression of MELK remarkably facilitated the migration, invasion and metastasis of GC cells (8); Vice versa, inhibition of MELK by shRNA or pharmacology inhibitor significantly suppressed GC cell migration and invasion, and inhibited the peritoneal spreading and metastasis in nude mice (8). Additionally, MELK depletion also greatly downregulated the expression of mesenchymal markers such as N-cadherin, Vimentin and Snail, but upregulated epithelial marker E-cadherin expression, suggesting targeting MELK suppressed the process of GC cell epithelial–mesenchymal transition (EMT), which is an important step for the initiation of tumor metastasis (17). However, Cheng et al. reported that knocking down MELK in the presence of TGF-β promoted EMT and cell migration in the lung cancer A549 cells (48). These data suggested that the role of MELK in tumor cell metastasis may vary in different types of human cancer.

In summary, this study demonstrated that MELK was highly expressed in ESCC and played an important role in regulating cancer development and progression. Mechanistically, we demonstrated that high expression of MELK may function to promote ESCC cell growth and metastasis at least in part by aberrantly activating FOXM1 signaling pathway. Collectively, these findings indicated MELK is a potential therapeutic target and small molecule inhibitors targeting MELK might be effective for the treatment of ESCC patients, even among those patients with advanced-stage disease.

## Data Availability Statement

All datasets generated for this study are included in the article/Supplementary Material.

## Ethics Statement

The studies involving human participants were reviewed and approved by the Ethics Committee of the First Affiliated Hospital of Henan University. The patients/participants provided their written informed consent to participate in this study. The animal study was reviewed and approved by the Henan University Institutional Animal Care and Use Committee.

## Author Contributions

LC performed and analyzed the experiments, and wrote the manuscript. QW and SB helped to conduct some experiments including Western blotting analysis and IHC staining. SX supported and directed the project and revised the manuscript. All authors read and approved the final manuscript.

### Conflict of Interest

The authors declare that the research was conducted in the absence of any commercial or financial relationships that could be construed as a potential conflict of interest.
