# Supplementary material for: Maternal Embryonic Leucine Zipper Kinase Promotes Tumor Growth and Metastasis via Stimulating FOXM1 Signaling in Esophageal Squamous Cell Carcinoma
Source: Front Oncol. 2020 Jan 28;10:10. doi: 10.3389/fonc.2020.00010 (PMC6997270; doi:10.3389/fonc.2020.00010)
Supplement: Supplementary file 1 [file Data_Sheet_1.PDF]

Supplementary Figure S1

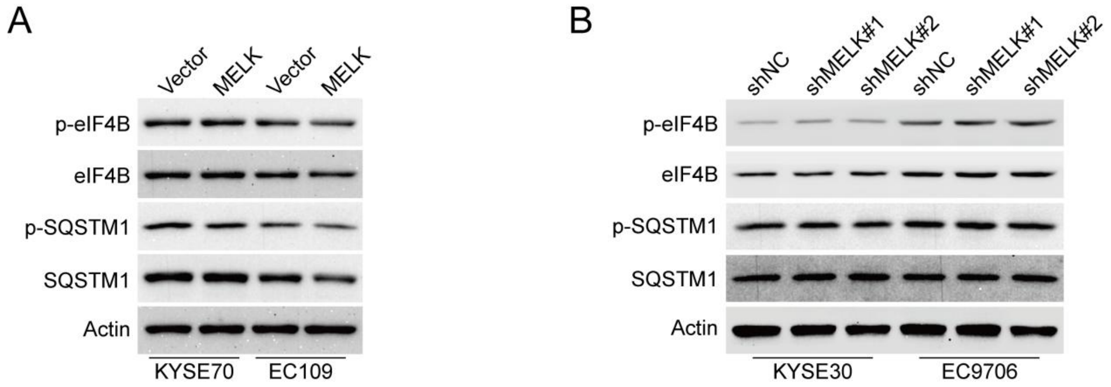

Figure S1. MELK has no effect on the phosphorylation of eIF4B and SQSTM1. Western blotting analysis was used to determine the protein levels of p-eIF4B (Ser406), eIF4B, p-SQSTM1 (Thr269/Ser272) and SQSTM1 in MELK-overexpressing KYSE70 and EC109 cells **(A)** and in MELK-depleted KYSE30 and EC9706 cells **(B)**. Actin served as an internal control.
