# Supplementary material for: Maternal Embryonic Leucine Zipper Kinase Promotes Tumor Growth and Metastasis via Stimulating FOXM1 Signaling in Esophageal Squamous Cell Carcinoma
Source: Front Oncol. 2020 Jan 28;10:10. doi: 10.3389/fonc.2020.00010 (PMC6997270; doi:10.3389/fonc.2020.00010)
Supplement: Supplementary file 2 [file Data_Sheet_2.PDF]

Supplementary Table S1

Table S1. Primers used for shRNA.

Gene	Sequence (5' to 3')
**shNC (No target shRNA)**	CCGGGCGCGATAGCGCTAATAATTTCTCGAGAAATTATTAGCGCTATCGCGCTTTTT
**shMELK#1**	CCGGCAGAAACAACAGGCAAACAATCTCGAGATTGTTTGCCTGTTGTTTCTGTTTTT
**shMELK#2**	CCGGGCCTGAAAGAAACTCCAATTACTCGAGTAATTGGAGTTTCTTTCAGGCTTTTT
**shFOXM1#1**	CCGGGCCCAACAGGAGTCTAATCAACTCGAGTTGATTAGACTCCTGTTGGGCTTTTT
**shFOXM1#2**	CCGGGCCAATCGTTCTCTGACAGAACTCGAGTTCTGTCAGAGAACGATTGGCTTTTT
